# Editorial Correspondence

**Published:** 1860-03

**Authors:** 


### EDITORIAL AND MISCELLANEOUS.

### Editorial Correspondence.

PHILADELPHIA, Jan. 26th, 1860.

*Dear Doctor:* I visited Blockley Hospital this morning, and witnessed a resection of the knee-joint by Dr. S. D. Gross. The subject was a woman twenty or thirty years of age, and apparently in good health. The leg was flexed upon the thigh with anchylosis of the knee-joint. The operation was for the relief of this unpleasant deformity, and to restore, to the extent possible, the *functions* of the limb.

The articulation was exposed by a semi-circular flap, the base extending from one condyle to the other—Dr. Gross' favorite method. It was only found necessary to remove the patella and a portion of the condyles, to extend the leg without difficulty. The wound was closed with the silver suture, and the limb placed upon a splint.

I was pleased to see that Dr. Gross used the silver suture in general Surgery. I regard it as an evidence of a move in the right direction. The silver suture has not been, nor is it now, in such favor here as in some other localities. It is strange, but true, that by some in the city, it is almost entirely ignored, as they do not use it even in operations where all are forced to admit that it has many advantages over the silk or linen suture. There appears to have been, and is still to some extent, a prejudice against its use, but from what cause I am unable to say. I certainly cannot give credence to the idea suggested by some, that nothing at first meets with favor in Philadelphia, that is not a Philadelphia discovery, or did not come from "across the waters." It is necessary to say, however, that there are not a few in Philadelphia who appreciate the advantages of the metallic suture, and use it when indicated.

I had the pleasure of meeting at Blockley, this morning, several distinguished medical gentlemen, who had visited the Hospital for the purpose of witnessing the resection above alluded to. Among others, was Dr. Levis, one of the editors of the "Medical and Surgical Reporter." I very much regret that I did not see more of Dr. Levis, as I regard him as one of the most agreeable and intellectual medical gentlemen that I have had the pleasure of meeting in the city.

Blockley is the largest charity in Philadelphia—perhaps the largest in the United States. It is certainly an immense establishment, with the greatest variety of disease; but notwithstanding this and other advantages claimed in connection with this Institution, there are not one hundred students of the twelve or fourteen hundred now in Philadelphia, that attend the two clinical lectures now being delivered each week, and comprising the only days that the Hospital is opened to students. This morning was an extra occasion at Blockley, it being generally known among the students that Dr. Gross was to resect the knee-joint, and, yet, there was not one hundred present to witness the operation. I am every day more and more convinced that Hospital instruction, as at present attempted in connection with the regular collegiate course, is but little more than a farce. I would not be understood to say that I regard Hospital instruction as a farce, or unimportant. Just the reverse is true; I regard it of the very greatest importance, but must be allowed to enter my protest against the plan at present adopted. That there is some radical defect, no one, who has for a moment seriously thought of the subject, will doubt. But, more of this when I reach New York, and see the result in the Hospitals of that great metropolis.

Very Respectfully,

Your Friend,

W. F. WESTMORELAND.

---

NEW YORK, Feb. 4th, 1860.

*Dear Doctor:* I reached New York several days ago, and since my arrival have been constantly on the tramp. I find that in the week or ten days that I have set apart to spend here, that I must be constantly going, if I see the half that would interest me in this "great medical metropolis." This, I hope, will be a sufficient apology for my apparent negligence in complying with my many promises, and also for the many imperfections that you will certainly find in my hurried letters.

The first medical man that I met after reaching the city, was that great and good man, Dr. J. Marion Sims, of the Woman's Hospital. I certainly can endorse with a right good will, every word you said in praise of Dr. Sims in your notice of your visit to New York and Philadelphia. I regard him as one of the greatest men of the age, and one who has certainly done more for the advancement of the surgical art, than any man of his day. If there was the least doubt of the very great worth of his contributions to surgery, to be convinced of their importance, it would only be necessary to refer to the effort now being made, both at home and abroad, to rob him of his well earned honors.

Through the courtesy of Dr. Sims, I have made the acquaintance of a number of distinguished medical gentlemen, and, among others, the father of American Surgery, Dr. Valentine Mott; one who has done more to give position to, and cause to be respected in Europe, American Surgery, than any man living. Dr. Mott, if I am not mistaken, is the rise of seventy years of age; still, no one to hear him lecture, or see him operate, (and I had the pleasure of both), would think him so old by half a score or more of years.

I to-day had the pleasure of spending an hour or two with that much abused man, Dr. Horace Green. He was kind enough to give me ample opportunity to witness his treatment of diseases of the air passages. I saw him operate upon several patients. In one, he injected the right bronchia with a solution of the nitrate of silver, and strange to say, it did not induce the least cough. The patient expressed herself as not feeling the least unpleasantness immediately after the operation. Dr. Green assures me that he does not propose to inject the solution of the nitrate of silver into a cavity of the lung, as has been alleged, and thus cure consumption, but bases his treatment upon the theory for which he strenuously contends, that the deposit of tubercles in the lungs, is always preceded by an irritation or an inflammation of the air passages or throat—most frequently a follicular inflammation of the throat, and that the deposit of tuberculous matter in the lung, is the result of the extension of the irritation. He does not rely upon the local treatment alone, but gives his patients such constitutional treatment as appears to be necessary to build up the general health. While we cannot agree with Dr. Green in all his positions, nor can we believe he has always pursued a proper course, still we do feel that he has been greatly misrepresented, and often persecuted without any just cause.

I had the pleasure, last night, of being present at a meeting of the Academy of Medicine, but am sorry to say that this learned body did not come up to my expectations. It is true that, from the inclemency of the weather, the meeting was sparsely attended, and perhaps other circumstances tended to detract from the interest of the meeting, but with due allowance for all this, the body did not present that dignified bearing, one would expect from a body of savans, representing a great "medical metropolis." The subject before the Academy, for discussion, was Diptheria. But few members took part in the discussion. There is certainly one glaring defect in the regulations of the Academy, which must strike every one as decidedly objectionable· I allude to the plan of reporting the proceedings of the Academy for the daily papers. Although every fellow of the Academy, would be indignant at seeing in the Herald or Tribune, a report by Physicians in the city, of a number of cases of a prevailing disease, successfully treated, still, all are content to see in the same daily papers, three or four hundred cases of Diptheria successfully treated by a fellow of the Academy, so the report reaches the secular papers through the medium of the Academy. This I think in bad taste.

Very Respectfully,

Your Friend,

W. F. WESTMORELAND.

---

NEW YORK, Feb. 8th, 1860.

*Dear Doctor:* I did not, I believe, in my last letter, speak of the Medical Schools and Hospitals of this "great medical centre." The schools here claim to be in a flourishing condition. There are between six and seven hundred students now in New York—not more than half the number that is in Philadelphia; still, the schools here have larger classes than ever before. The University of New York has something over four hundred Matriculates, the College of Physicians and Surgeons about two hundred, and the New York Medical College between fifty and an hundred. They claim to have lost no Southern students during the excitement some weeks ago, yet I learn from a reliable source, that there were a few who left to join the party leaving Philadelphia.

If Philadelphia is far in advance in the way of Medical Schools and large classes, New York can certainly boast of her superior Hospitals and Hospital regulations. The City Hospital, on Broadway, and Bellevue, on East River, the two central institutions, cannot be excelled in any country. They each have between five and six hundred beds, which are neat and well arranged. The only defect that I have noticed in their regulations, is the frequent change of the Medical and Surgical staff. There is a succession every two months. The attending physician, or surgeon, has only time to become well established and interested in his duties, when he has to give place to his successor. It is certainly often very annoying to those on service, as well as to the patients and those following the service. A change once in six months is certainly sufficiently often. The unpleasant effects, resulting from this regulation, were, to me, made very striking, as a change occurred only a day or two after my arrival in the city.

In a letter from Philadelphia, I spoke of the perfect failure in that city, of Hospital instruction in connection with the regular course, and promised to refer to the subject again, after reaching New York and noting the result here.

There is no denying the fact that the Hospital advantages offered the student here, are greatly superior to those in Philadelphia; still I do not see that the student is to any great extent profited by these advantages. The student here is admitted to the wards of the Hospital, and may, if he is disposed, attend the regular visit each day. I have, however, visited the City Hospital several times since my arrival, and notwithstanding the privileges above alluded to, I did not meet half a dozen students either day. There is a clinical lecture twice a week, in each of the Hospitals, with operations, &c., as in Philadelphia, but as I did not have an opportunity of visiting the Hospitals during these exercises, I cannot speak of the attendance.

The truth is, students have no time to visit the Hospitals regularly, if they were disposed. With the seven branches usually taught in medical schools, crowded into a four month's course, with from five to seven lectures a day, the student has but little time for Hospital instruction. Each school here has its regular College Clinics, as in Philadelphia, but no one that I have yet attended, comes up to the Clinic of the Jefferson Medical College. The only plan to have students attend the Hospitals regularly, is to have a course of instruction in the Hospitals, unconnected with the Collegiate course, with the power to confer the degree after they have graduated in medicine.

Through the courtesy of Dr. Sims, I had the pleasure, a few days ago, of spending several hours at the Woman's Hospital. Aside from the pleasure of examining a number of interesting cases, I witnessed two operations for vesico-vaginal fistula. Dr. Sims is certainly a fine operator. He makes his incisions and passes his sutures, apparently, with the same facility and rapidity that he would were the parts more directly under his control. No one need ever hope to excel him in this, his favorite operation. He has entirely abandoned his clamps and everything else, but the simple silver suture, which he assures me he finds entirely sufficient. This I was pleased to learn, as my own limited experience has long since convinced me that the numerous plans proposed to fasten the sutures, as the button, bors, clamps, &c., &c., have no real advantages, and often seriously complicate the operation.

The day spent at the Woman's Hospital, fully repaid me for my visit to New York—had I have seen nothing else, I should *never* have regretted my trip.

I met, to-day, the veteran editor of the American Medical Gazette, Dr. D. Meredith Reese, a man of noble heart and towering intellect. Dr. Reese is unconnected with any medical school, and stands aloof from all cliques. He is a bold and fearless writer, as all will testify who read that best of medical journals, the American Medical Gazette.—He does not fear to condemn wrong whenever found, and is equally willing to do justice to all. Long may he live to defend that noble profession of which he has been an ornament.

I spent the day yesterday, with Dr. Lewis Sayre, one of the Surgeons to Bellevue Hospital. Dr. Sayre has for some years, been giving special attention to diseases of the joints, and in their treatment has certainly met with better success than is usually experienced in this troublesome class of diseases. He did me the kindness to show me a number of his private patients; the majority being diseases of the joints. The result of treatment in several of the cases, was truly surprising. One of the prominent features in Doctor Sayre's treatmeut of this class of affections, in the second and third stages, is not to permit the accumulation of pus in or around the articulation. He does not hesitate to make free incisions into the joint itself, and even to pass a seton through the diseased articulation, if it is thought necessary or its perfect drainage. He contends, and justly, too, that no reporation can take place while the tissues composing the articulation are constantly bathed in pus. I saw two cases where the patients had suffered greatly from a strumuous disease of the ankle joint, in which Dr. Sayre had not only made free incisions, but in both had passed setons through the ankle joints, and then suffered them to remain for two or three months, or until suppuration had to a great extent subsided; and strange to say, one of the patients recovered with but little deformity of the ankle joint, and perfect motion; and the other with a partial destruction of the astragalus, with still some motion in the joint. In the first stage of hip-joint disease, or in a similar disease in other joints, he takes off the pressure from the inflamed serous membrane by a section of the muscles, which may be found tense, and by extension and counter extension. Dr. Sayre was the first surgeon in America who resected the hip-joint; and I had the pleasure yesterday, of examining the young lady upon whom it was performed. The operation was performed five or six years ago; she is now fifteen or sixteen years of age, and in perfect health. The limb is shortened between two and three inches; she has, however, perfect control over its motions—can adduct, abduct, extend and flex the limb with the same ease apparently, as upon the opposite side.

I have had the pleasure of meeting your friend, Dr. John C. Dalton, jr., the "*great Physiologist*" of America. He has placed me under great obligations for attentions extended during my visit. He certainly occupies an enviable position as a Physiologist.

I very much regret, that from circumstances unavoidable, I have not seen more of that eminent surgeon, Dr. J. M, Carnochan, Prof. of Surgery in the New York Medical College, and Surgeon-in-Chief to the State Emigrants' Hospital. Although he is comparatively a young man, I regard him as one of the first surgeons in New York.

Before closing this hurried letter, I must be permitted to again extend my many thanks to Dr. J. Marion Sims, as I feel that for the greater portion of the pleasure derived from this visit to New York, I am indebted to his kind attentions.

I leave to-night for Philadelphia, from which place you may hear from me again.

Most respectfully yours, &c.,
W. F. WESTMORELAND.

